# Lobectomy with bronchoplasty and pulmonary arterial angioplasty for lung cancer after correction of contralateral partial anomalous pulmonary venous connection

**DOI:** 10.1186/s40792-020-01083-6

**Published:** 2020-12-07

**Authors:** Koichi Fukumoto, Madoka Goto, Yasuhisa Ichikawa, Yuta Kawasumi, Mika Uchiyama, Atsuo Maekawa, Shoichi Mori

**Affiliations:** 1 Department of Thoracic Surgery, Japanese Red Cross Nagoya First Hospital, 3-35 Michishita-cho, Nakamura-ku, Nagoya, 453-8511 Japan; 2 Department of Cardiovascular Surgery, Fujita Health University School of Medicine, 1-98 Dengakugakubo, Kutsukake, Toyoake, Aichi 470-1192 Japan

**Keywords:** Lung cancer, Partial anomalous pulmonary venous connection, Bronchoplasty, Pulmonary arterial angioplasty

## Abstract

**Background:**

There have been few reports on surgically treated primary lung cancer accompanied by contralateral partial anomalous pulmonary venous connection (PAPVC). In such cases, repair of the PAPVC might be necessary to avoid postoperative right-heart failure due to the increased flow of the left-to-right shunt.

**Case presentation:**

We herein report a case of lung adenocarcinoma treated by left-upper lobectomy with bronchoplasty and pulmonary arterial angioplasty after induction chemoradiation therapy followed by surgical correction of the PAPVC in the right-upper lobe. The patient is alive without recurrence of lung cancer or any symptoms of heart failure 17 months after pulmonary resection.

**Conclusion:**

When considering performing major pulmonary resection for lung tumor, thoracic surgeons should pay close attention to the presence of a PAPVC not only on the ipsilateral side of the lung tumor, but also the contralateral side, although it is a rare phenomenon.

## Background

Minor anatomical congenital anomalous conditions, such as pulmonary artery, pulmonary vein and bronchus, are mostly asymptomatic and rarely cause serious problems after pulmonary resection. However, when vascular shunt is present in another lobe of the lung, anatomical pulmonary resection may cause fatal problems owing to the increase in vascular shunt. Partial anomalous pulmonary venous connection (PAPVC), which is the same condition as partial anomalous pulmonary venous return (PAPVR), is a relatively rare congenital anomaly with an incidence of 0.4–0.7% in the general population [1]. Surgical repair of a PAPVC is recommended in patients with symptoms and/or an elevated pulmonary-to-systolic blood flow ratio (Qp/Qs).

When pulmonary resection is scheduled in patients with lung tumor and a PAPVC, not only Qp/Qs, but also the location of the PAPVC and lung tumor is important. In patients with a PAPVC and lung tumor located in the same lobe, lobectomy with ligation of the anomalous vein can be the definitive treatment for both the PAPVC and lung tumor. However, when a PAPVC and lung tumor are located in different lobes, correction of the PAPVC might be necessary to avoid postoperative right-heart failure due to the increased flow of the left-to-right shunt.

We herein report the successful treatment of a 66-year-old woman with left lung adenocarcinoma by left-upper lobectomy with bronchoplasty and pulmonary arterial angioplasty after surgical correction of contralateral PAPVC.

## Case presentation

**Fig. 1:**
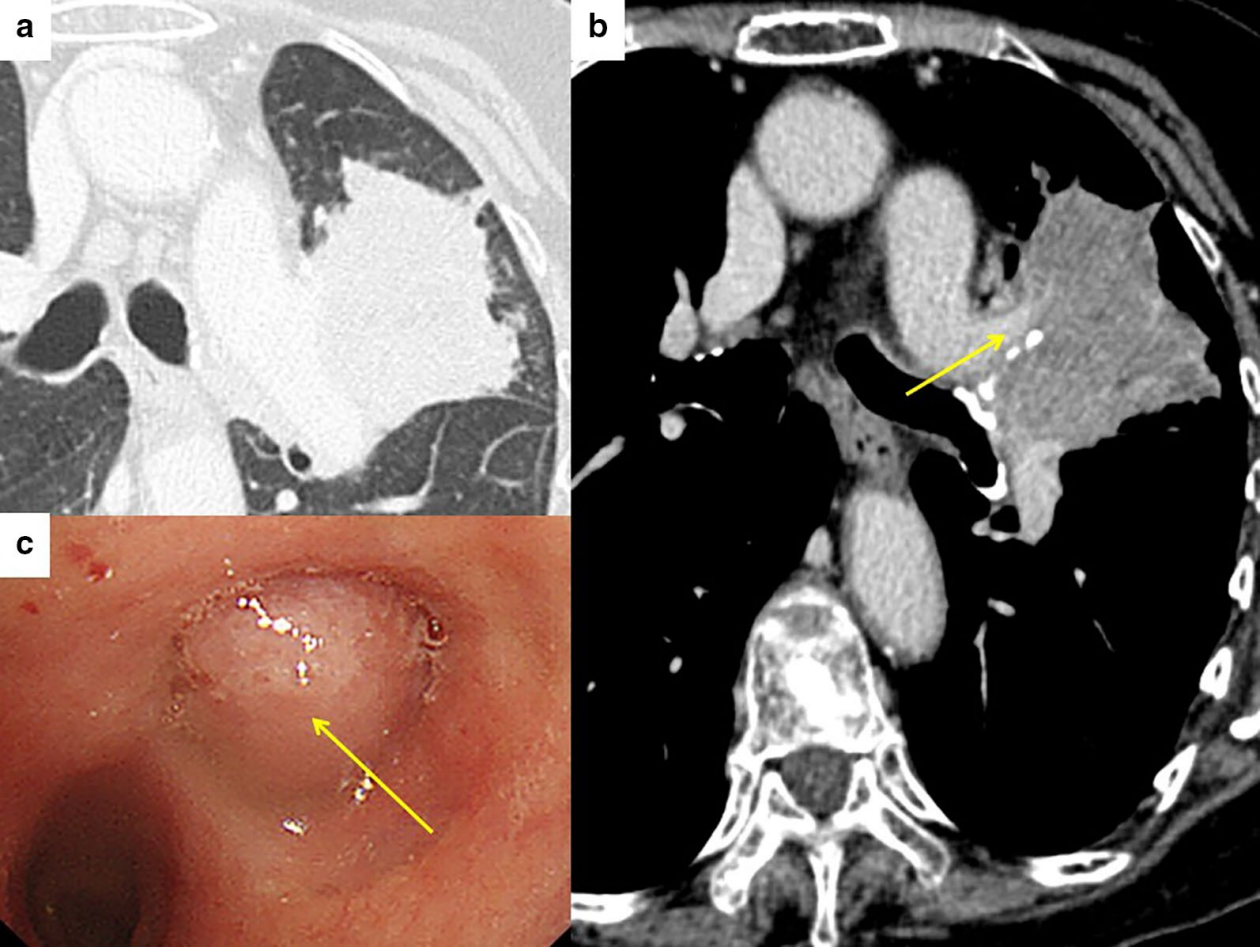
Pre-treatment enhanced chest computed tomography showed a solid mass measuring 6.2 cm in maximal diameter in the left-upper lobe (**a**) accompanied by narrowing of the left-upper lobe pulmonary artery surrounded by the mass (**b**). Bronchoscopy findings showed that the left-upper bronchus was completely obstructed by the polypoid tumor (**c**)

**Fig. 2:**
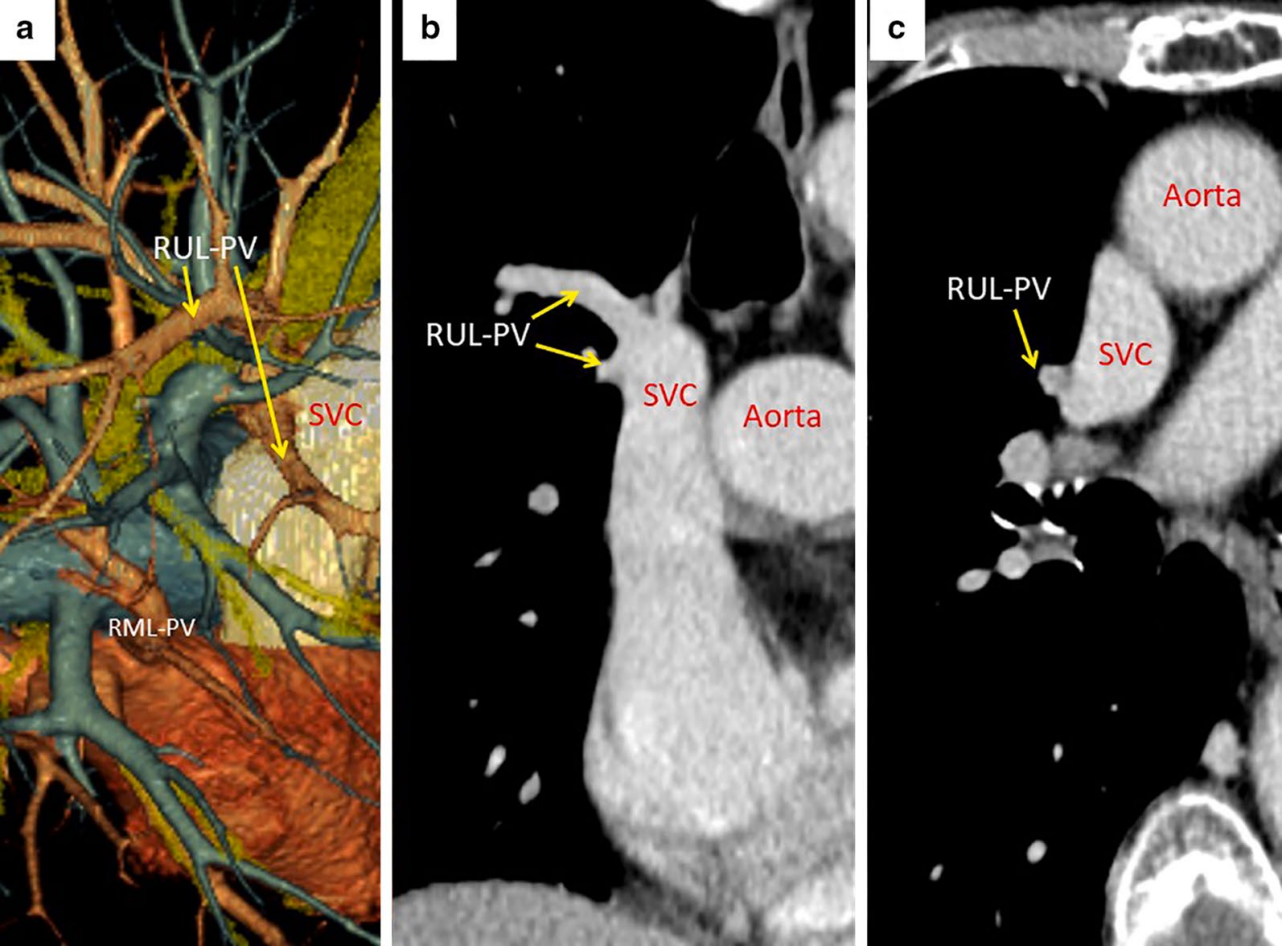
Three-dimensional computed tomography (CT) angiography (**a**) and enhanced CT (**b**: coronal view, **c**: axial view) revealed the partial anomalous pulmonary venous connection in the right-upper lobe returning into the superior vena cava (SVC). *RUL* right-upper lobe, *RML* right-middle lobe, *PV* pulmonary vein

A 66-year-old woman complaining hemoptysis was referred to our hospital. Enhanced chest computed tomography (CT) showed a solid mass measuring 6.2 cm in maximal diameter in the left-upper lobe (LUL) (Fig. 1a, b) accompanied by the narrowing of the LUL pulmonary artery and bronchus surrounded by the mass. Bronchoscopy findings showed that the left-upper bronchus was completely obstructed by the polypoid tumor (Fig. 1c). Three-dimensional CT angiography (Fig. 2a) and enhanced CT (Fig. 2b: coronal view, Fig. 2c: axial view) revealed a PAPVC in the right-upper lobe (RUL) returning into the superior vena cava (SVC).

The mass was pathologically confirmed to be non-small cell lung cancer (NSCLC) by a transbronchial lung biopsy. Her disease was diagnosed as cT3N1M0, stage IIIA, NSCLC. Transthoracic echocardiography showed no evidence of other cardiovascular abnormalities, such as atrial septal defect (ASD).

**Fig. 3:**
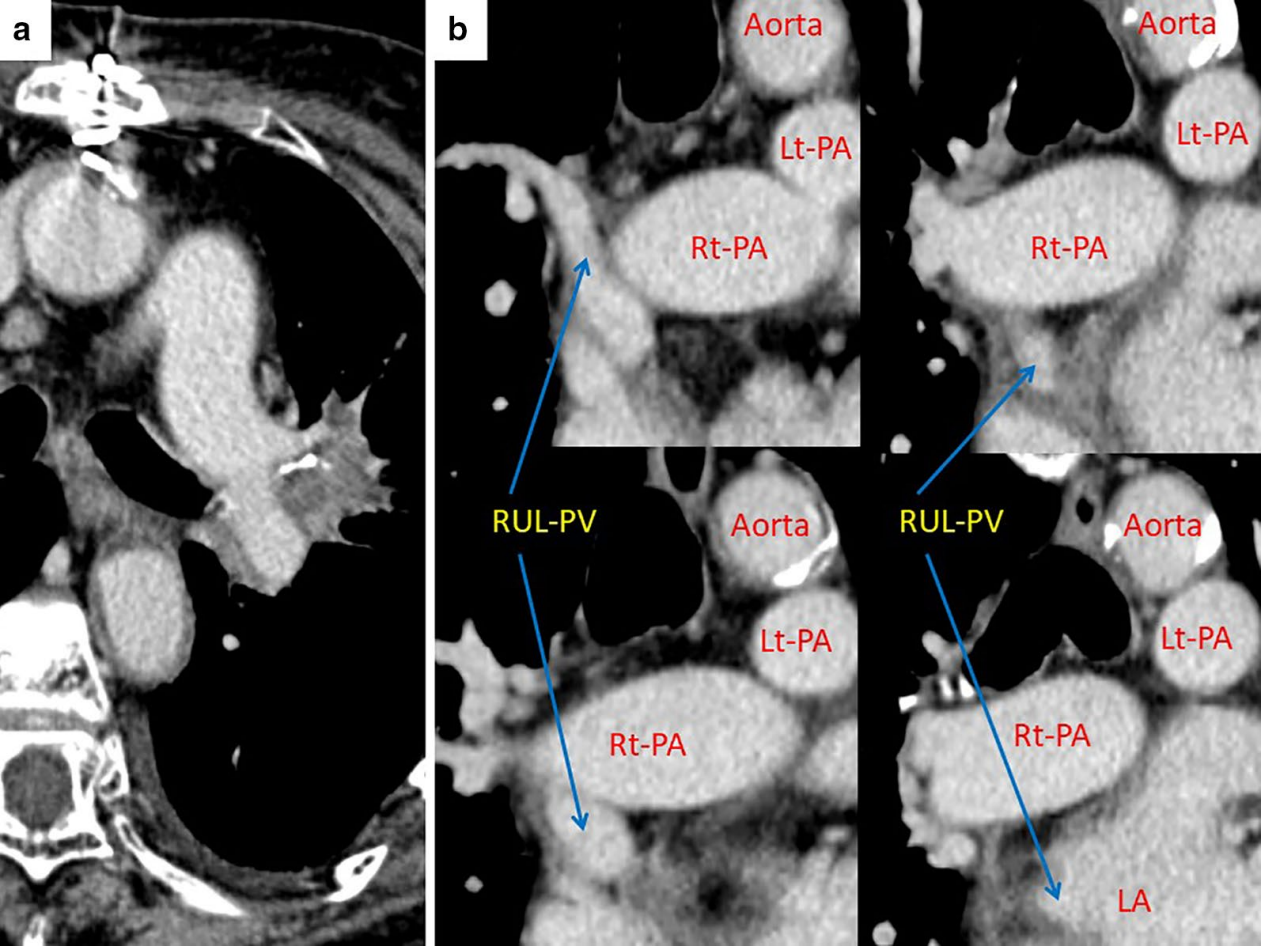
Enhanced chest computed tomography after induction chemoradiation therapy (**a**) revealed a 3.2-cm solid mass in the left-upper lobe that showed a partial response according to the response evaluation criteria in solid tumor (− 49% in size). The enhanced chest computed tomography (coronal view) after surgical correction of the partial anomalous pulmonary venous connection in right-upper lobe (**b**) showed the right-upper lobe pulmonary vein draining into the left atrium. *RUL* right-upper lobe, *RML* right-middle lobe, *PV* pulmonary vein, *PA* pulmonary artery, *Rt* right, *Lt* left

**Fig. 4:**
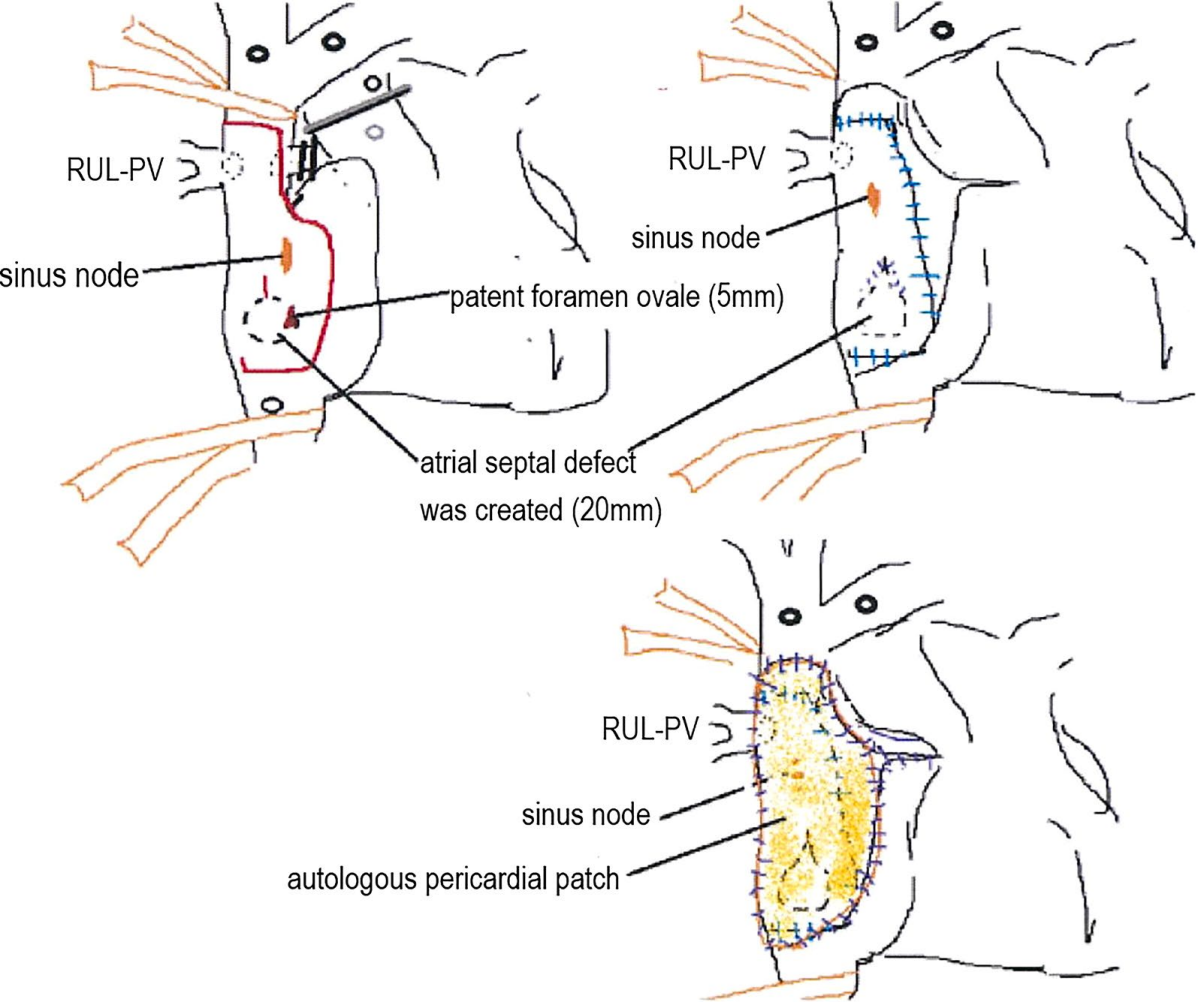
Surgical schema of the correction of partial anomalous pulmonary venous connection (PAPVC) in this patient is shown. *RUL-PV* right-upper lobe pulmonary vein

**Fig. 5:**
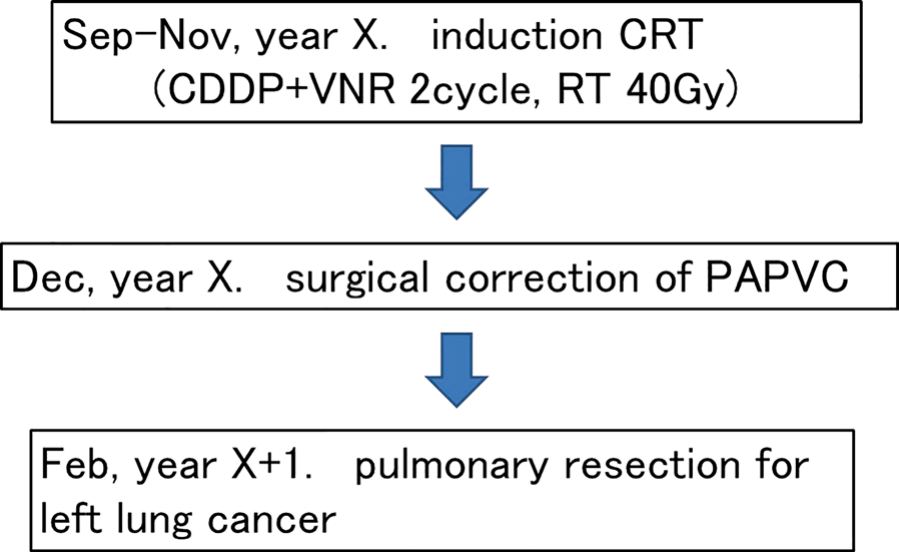
The time schedule from induction chemoradiation therapy to pulmonary resection in this patient is shown. *CRT* chemoradiation therapy, *CDDP* cisplatin, *VNR* vinorelbine, *RT* radiotherapy, *PAPVC* partial anomalous pulmonary venous connection

A pre-treatment cardiac catheterization test showed the Qp/Qs to be 2.24. To avoid fatal right-heart failure after pulmonary resection, we decided to perform correction of the PAPVC in the RUL prior to pulmonary resection. After induction chemoradiation therapy (2 cycles of cisplatin plus vinorelbine with concurrent 40-Gy radiotherapy), the LUL mass showed a partial response according to the response evaluation criteria in solid tumor (Fig. 3a: 3.2 cm in largest diameter, − 49% in size). Correction of the PAPVC in the RUL was performed by cardiovascular surgeons under cardiopulmonary bypass (double-decker method) via median sternotomy [2]. Figure 4 shows the surgical schema of the correction of PAPVC in this patient. The total operation time and the pump time was 259 min and 143 min. The patient required 8 units of red blood cell transfusion intraoperatively. The enhanced chest CT (coronal view) after surgical repair of the PAPVC in the RUL (Fig. 3b) showed the RUL pulmonary vein draining into the left atrium. Thereafter, left-upper lobectomy with bronchoplasty and pulmonary arterial angioplasty was performed. Figure 5 shows the time schedule from induction chemoradiation therapy to pulmonary resection in this patient.

The postoperative course was uneventful except for paroxysmal atrial fibrillation. A pathologic examination of the resected specimen showed that almost 50% of solid predominant adenocarcinoma cells were viable (ypT1cN1M0, stage IIB) with a negative bronchial margin. After pulmonary resection, the patient received no adjuvant therapy. The patient remains alive without recurrence of disease or symptoms of heart failure 17 months after pulmonary resection. So far, the patient also continues to take anti-coagulation therapy (warfarin) after the surgical correction of PAPVC.

## Discussion

PAPVC is a relatively rare congenital anomaly often associated with other congenital heart diseases, such as ASD, in children [3]. A recent radiologic study in adults showed the disease prevalence to be 0.1%, with a mean patient age of 58 years old and a 58% female predominance [4]. The most common location was the left-upper lobe PV draining into the left brachiocephalic vein. One of the surgical indications reported by Hijii et al. is a Qp/Qs greater than 2.0 [5].

When considering major pulmonary resection for lung tumor in patients with PAPVC, the location of the lung tumor and PAPVC is extremely important, as pulmonary resection without correction of the PAPVC might lead to right-heart failure. In the present case, the PAPVC was located in the RUL, while the lung cancer was located in the LUL. We decided to perform correction of the contralateral PAPVC before pulmonary resection in order to avoid postoperative fatal right-heart failure.

Although several cases of lung tumor with PAPVC have been reported, surgically managed pulmonary tumors with contralateral PAPVC are extremely rare. Black et al. reported a case of right pneumonectomy for lung cancer accompanied by contralateral PAPVC that was detected after pulmonary resection. Unfortunately, this patient died of fatal right-heart failure [6]. Sakurai et al. reported successful surgical repair of right PAPVC under cardiopulmonary bypass followed by left pneumonectomy for left lung cancer [7]. To our knowledge, the present case is the first of lobectomy with bronchoplasty and pulmonary arterial angioplasty for lung cancer after correction of a contralateral PAPVC. While there is a chance of detecting ipsilateral PAPVC intraoperatively [8], it would be impossible to detect contralateral PAPVC intraoperatively.

## Conclusions

When considering performing major pulmonary resection for lung tumor, thoracic surgeons should pay close attention to the presence of a PAPVC not only on the ipsilateral side of the lung tumor, but also the contralateral side, although it is a rare phenomenon.

## Data Availability

Data sharing is not applicable to this article as no datasets were generated or analyzed during the current study.

## Abbreviations

PAPVC: Partial anomalous pulmonary venous connection
PAPVR: Partial anomalous pulmonary venous return
Qp/Qs: Pulmonary-to-systolic blood flow ratio
CT: Computed tomography
LUL: Left-upper lobe
RUL: Right-upper lobe
SVC: Superior vena cava
NSCLC: Non-small cell lung cancer
ASD: Atrial septal defect
